# Announcement: 4th International Conference on Environmental and Biological Aspects of Main-Group Organometals (ICEBAMO 98)

**DOI:** 10.1155/MBD.1997.300

**Published:** 1997


					b
ICEBAMO 98
4TH INTERNATIONAL CONFERENCE ON ENVIRONMENTAL AND
BIOLOGICAL ASPECTS OF MAIN-GROUP ORGANOMETALS
Odense, Denmark, 28 June to 2 July, 1998
The ICEBAMO meetings aim to facilitate interdisciplinary discussion on environmental and biological
aspects of main-group organometals. Previous meetings in Padua (1986 and 1991) and Bordeaux
(1994) have brought together researchers from the fields of toxicology, environmental sciences,
analytical chemistry, biochemistry, and synthetic organometallic chemistry. ICEBAMO 98 in Odense
will provide a forum for discussing the origin, behaviour and fate of organometallic compounds in the
environment, and their application to and impact on biological systems. Focus will be on the
following aspects of organometallic compounds:
Origin and pathways in the environment
Fate in organisms including accumulation and toxicological effects
Biological and biochemical studies on transformation/detoxification processes
Analytical chemistry including chemical speciation techniques
Main group metal-based drugs
The meeting will consist of oral presentations of invited and contributed papers, and poster
presentations. Suitable manuscripts will be published in Applied Organometallic Chemistry or Metal-
Based Drugs as appropriate following the usual refereeing process.
Additional and updated information may be obtained from the
ICEBAMO98
homepagehttp:llwww.ou.dklnatlbiologylicebamo.html
or by contacting
Kevin Francesconi,
Institute of Biology, Odense University,
5230 Odense M, Denmark.
Fax" (45) 65 93 04 57. E-mail" kaf@biology.ou.dk
300

				

